# Development and External Validation of a Novel Immune Checkpoint–Related Gene Signature for Prediction of Overall Survival in Hepatocellular Carcinoma

**DOI:** 10.3389/fmolb.2020.620765

**Published:** 2021-01-21

**Authors:** Enfa Zhao, Shimin Chen, Ying Dang

**Affiliations:** ^1^Department of Structural Heart Disease, The First Affiliated Hospital of Xi'an Jiaotong University, Xi'an, China; ^2^Department of Gastroenterology, Traditional Chinese Medicine Hospital of Taihe Country, Taihe, China; ^3^Department of Ultrasound Medicine, The First Affiliated Hospital of Xi'an Jiaotong University, Xi'an, China

**Keywords:** hepatocellular carcinoma, immune checkpoint, signature, overall survival, database

## Abstract

**Objective:** The purpose of this study was to develop and validate a novel immune checkpoint–related gene signature for prediction of overall survival (OS) in hepatocellular carcinoma (HCC).

**Methods:** mRNA expression profiles and clinical follow-up information were obtained in the International Cancer Genome Consortium database. An external dataset from The Cancer Genome Atlas (TCGA) Liver Hepatocellular Carcinoma database was used to validate the results. The univariate and multivariate Cox regression analyses were performed based on the differentially expressed genes. We generated a four-mRNA signature to predict patient survival. Furthermore, the reliability and validity were validated in TCGA cohort. An integrated bioinformatics approach was performed to evaluate its diagnostic and prognostic value.

**Results:** A four-gene (epidermal growth factor, mutated in colorectal cancer, mitogen-activated protein kinase kinase 2, and NRAS proto-oncogene, GTPase) signature was built to classify patients into two risk groups using a risk score with different OS in two cohorts (all *P* < 0.0001). Multivariate regression analysis demonstrated the signature was an independent predictor of HCC. Furthermore, the signature presented an excellent diagnostic power in differentiating HCC and adjacent tissues. Immune cell infiltration analysis revealed that the signature was associated with a number of immune cell subtypes.

**Conclusion:** We identified a four–immune checkpoint–related gene signature as a robust biomarker with great potential for clinical application in risk stratification and OS prediction in HCC patients and could be a potential indicator of immunotherapy in HCC. The diagnostic signature had been validated to accurately distinguish HCC from adjacent tissues.

## Introduction

Hepatocellular carcinoma (HCC), which is characterized by a low survival rate, aggressive nature, and high metastasis potential, is the most common subtype of hepatic malignancies worldwide, accounting for ~90% of primary liver cancers (Bray et al., 2018). There are ~841,000 new confirmed cases of HCC and 782,000 deaths in 2018. Although the great developments in radiotherapy, chemotherapy, liver transplantation, and other potentially curative treatment have revolutionized the treatment of HCC, the long-term prognosis remains poor because most HCC patients are at the late stage at the time of diagnosis and have lost the opportunity of surgical removal of their lesion (Bruix et al., 2014). Most patients with advanced-stage HCC ultimately do not benefit from traditional medications (Stravitz et al., 2008). Therefore, high-risk HCC patients with potentially poor prognosis must be monitored, and timely and effective treatment should be taken to prolong the survival and improve the quality of life (Llovet et al., 2015). Traditional methods utilizing clinical tumor-node-metastasis (TNM) staging, vascular invasion, and other clinicopathologic parameters contribute to predict HCC prognosis (Bruix et al., 2016). Despite the availability of multiple treatment opportunities, diagnosis is still made in an advanced stage, limiting application of most therapeutic choices that currently are based on the Barcelona Clinic Cancer Liver Classification system (Aho et al., 2014). However, considering the great complexity and heterogeneity of HCC, the predictive ability of such models is still far from satisfying.

In recent years, the emergence of immune checkpoint inhibitors has revolutionized the therapeutic landscape of cancer patients. Sorafenib was well-established as a standard of care for HCC for nearly a decade until 2018 that lenvatinib has finally become the first-line treatment in clinical practice, and regorafenib, ramucirumab, and cabozantinib have been recommended as second-line drugs approved by the US Food and Drug Administration (Longo et al., 2019; Dong et al., 2020). Recently, the seminal IMbrave150, a global, multicenter, open-label, phase 3 randomized trial, has approved of immunotherapy plus antiangiogenesis (atezolizumab combined with bevacizumab) for first-line systemic treatment for unresectable HCC in many countries (Finn et al., 2020). In the past 3 years, new data from trials of immune checkpoint inhibitors provided multiple new options for advanced HCC (Dong et al., 2020). It is well known that tumor cells can evade immune surveillance and promote cancer growth and progression by activating various immune checkpoint pathways. Programmed death (PD) ligand 1 (PD-L1)/PD1 and cytotoxic T-lymphocyte antigen (CTLA)-4 inhibitors have been used in multiple malignancies, whereas crucial molecules able to disturb other coinhibitory signaling pathways are under investigation (Longo et al., 2019). In the era of immunotherapy, immune checkpoint inhibitors have also been used for HCC patients; however, not all patients could benefit from immunotherapy (Miamen et al., 2012; Mahn et al., 2020). There is an urgent need for effective biomarkers in patients with HCC to improve survival prediction and early diagnosis and the potential benefits of immunotherapy. Therefore, based on the immune checkpoint–related genes, we used two cohorts to develop and validate a robust prognostic signature for HCC and explore its diagnostic value, as well as contribute to determine effective immunotherapy for HCC.

## Methods

### Data Collection and Immune Checkpoint–Related Gene Acquisition

Level 3 mRNA expression data and corresponding clinical follow-up information for 240 patients with primary HCC (231 with complete follow-up information) and 202 adjacent tissues were downloaded from the International Cancer Genome Consortium (ICGC) database (https://dcc.icgc.org/, LIRI-JP). RNA-sequencing data from 374 HCC patients and 50 adjacent tissues with corresponding clinical follow-up information (370 with complete follow-up information) were downloaded from The Cancer Genome Atlas (TCGA) database and were used for external validation of the signature. The probe IDs were changed into the corresponding gene symbols based on their annotation files. When several probes matched to an identical gene symbol, we averaged them for further analysis. The PD-1/PD-L1 and CTLA-4 signaling pathways genes were extracted from the KEGG [Kyoto Encyclopedia of Genes and Genomes (https://www.kegg.jp/)] and Reactome (https://www.reactome.org/) pathway database to retrieve crucial genes of the PD-1/PD-L1 and CTLA-4 signaling pathways. A total of 282 unique candidate genes were retrieved from KEGG (*n* = 225) and Reactome (*n* = 97) pathway database (Supplementary Table 1). The identified intersection sets among immune checkpoint–related genes and ICGC and TCGA cohorts were used for subsequent analysis.

### Prognostic Genes Identification and Gene Signature Construction

The differentially expressed genes (DEGs) between HCC tissues and adjacent tissues were screened using the “limma” R package with absolute value of the log2 fold change (logFC) >1 and false discovery rate (FDR) <0.05 in the ICGC cohort. Next, the relationship of DEGs with overall survival (OS) in HCC was calculated with univariate Cox regression analysis. We further narrowed the gene range in the univariate analysis with *P* < 0.05 by performing LASSO-penalized Cox regression analysis with 10-times cross-validations using the glmnet package in R. Multivariate analysis was finally used to identify the optimal model according to the smallest Akaike information criterion value, which is a measure of the goodness of fit (Aho et al., 2014). Afterward, the identified immune checkpoint gene-based prognosis risk score was designed on the basis of linearly combining the risk score formula with the expression level multiplied regression model (β). Risk score = βgene_1_^*^ gene_1_ expression + βgene_2_^*^ gene_2_ expression + … + βgene_n_^*^ gene_n_ expression. Therefore, a risk score was obtained for each patient based on the risk score formula. All the patients were classified into high-risk and low-risk groups based on the median value of the risk score as a cutoff value. Kaplan–Meier analysis was performed to compare the statistical differences in survival rate between the high-risk and low-risk groups. Time-dependent receiver operating characteristic (ROC) curve analysis and area under the curve (AUC) for 1-, 3-, and 5-year OS was carried out to determine the clinically predictive ability of the gene signature. Prognosis prediction performance was evaluated by the AUC for 1-, 3-, and 5-year OS from the time-dependent ROC analysis.

### Independence of the Prognostic Gene Signature

The univariate Cox regression analysis was performed to screen out the significance of the novel signature and clinicopathologic parameters on the OS of patients with HCC. Multivariate Cox regression analysis was further conducted to identify independent prognostic variables. Survival analysis was carried out to validate the risk stratification ability of the novel signature when patients were classified into different clinical subgroups.

### External Validation of Gene Expression Pattern and Prognostic Signature

TCGA cohort was used for the validation of identified DEGs. The risk score of each patient in the TCGA cohort was calculated based on the risk formula mentioned above, and patients were classified into the high- or low-risk groups according to the cutoff point of the median risk score. The same analyses were conducted to validate the reliability and validity of the novel signature, including Kaplan–Meier analysis, ROC curve analysis, and multivariate Cox proportional hazards analysis.

### Constructing and Validating a Predictive Nomogram

A composite nomogram was established based on all independent prognostic parameters identified by the multivariate Cox proportional hazards analysis to predict the probability of 1-, 3-, and 5-year OS using the “rms” package in R software. Validation of the nomogram was explored by discrimination and calibration. The calibration plot and the concordance index (C-index) were used to assess the performance of the prediction model by a bootstrap method in both cohorts. The decision curve analysis was carried out to explore the clinical effectiveness of the model in comparison with the AJCC staging system. The optimal model is the one with the highest net benefit as calculated.

### Construction and Validation of the Diagnostic Performance of the Immune Checkpoint–Related Gene Signature

To explore the diagnostic potential of the novel gene signature in distinguishing HCC patients from adjacent tissues, ROC analysis of each identified gene was performed between 240 patients with HCC and 202 adjacent tissues in the ICGC cohort and further validated in 374 HCC and 50 adjacent samples in the TCGA. Support vector machine (SVM) is a supervised classification model with widely acknowledged generalizability (Cherkassky, 1997). Therefore, we established a diagnostic classifier with identified immune checkpoint–related gene genes by using the SVM to distinguish HCC from adjacent tissues. Furthermore, the performance of the classifier in n distinguishing early stage of HCC patients (stage I) from adjacent tissues was further measured via the AUCs in both cohorts.

### Gene Set Enrichment Analysis

To explore the alerted biological processes underlying the new established prognostic signature, GSEA was carried out to investigate whether the identified sets of genes presented statistically significant differences between the high- and low-risk groups (Thomas et al., 2011). Gene sets at *P* < 0.05 and an FDR < 0.25 were considered to be significantly enriched and to identify biological processes.

### Immune Cell Subtypes and Its Correlation With Identified Immune Checkpoint–Related Genes

To investigate the relative abundance of tumor-infiltrating immune cells from gene expression profiles in HCC, the analytical tool called CIBERSORT (https://cibersortx.stanford.edu/) was used to calculate immune cell infiltrations. The algorithm estimated the putative abundance of immune cells using a reference set with 22 immune cell subtypes (LM22) with 1,000 permutations (Newman et al., 2015). We used the mRNA expression matrix as the input files to evaluate the immune infractions of each sample through the CIBERSORT algorithm (Zhao et al., 2020). Cases with a CIBERSORT output of *P* < 0.05, demonstrating that the inferred proportions of immune cell populations produced by CIBERSORT are accurate (Ali et al., 2016), were filtered out for subsequent analysis. The CIBERSORT output values were defined as immune cell infiltration fraction per sample. For each case, the sum of 22 immune cell type fractions equaled 1. The associations of the feature genes with infiltrating immune cells levels were investigated by Spearman rank correlation analysis using the R software and were visualized with “ggplot2” package.

### Analysis of Immunotherapy Efficacy in the Validation Cohort

Tumor mutation burden (TMB) can reflect the total number of mutations in cancer cells, which could be used for evaluating the therapeutic effect of immunotherapy (Liu et al., 2019). The mutation data of HCC patients were downloaded and stored as MAF format in the TCGA data portal. TMB analysis was performed by R package “maftools” (Mayakonda et al., 2018). The association between the risk score and expression levels of immune checkpoint genes (CTLA4, PD1, and PD-L1) was investigated.

### Statistical Analysis

The expression changes of identified genes between HCC and normal samples were compared using Student *t*-test. A heatmap was generated using the “pheatmap” package of the R software. Survival curves were generated using the “survival” package. The ROC curves were performed by an R package “survivalROC.” The “rms” package was used for nomogram construction, and the Hmisc package was used for calculation of C-index. Multivariate Cox proportional hazards regression analyses with 95% confidence intervals (CIs) were used to identify potential prognostic factors. The visualization of 22 types of infiltrating immune cells was performed by using R package “corrplot.” *P* < 0.05 was considered to be significant. All statistical analyses were performed using R (version 3.6.3; https://www.r-project.org/).

## Results

### Patient Demographics and Clinical Characteristics

**Figure 1:**
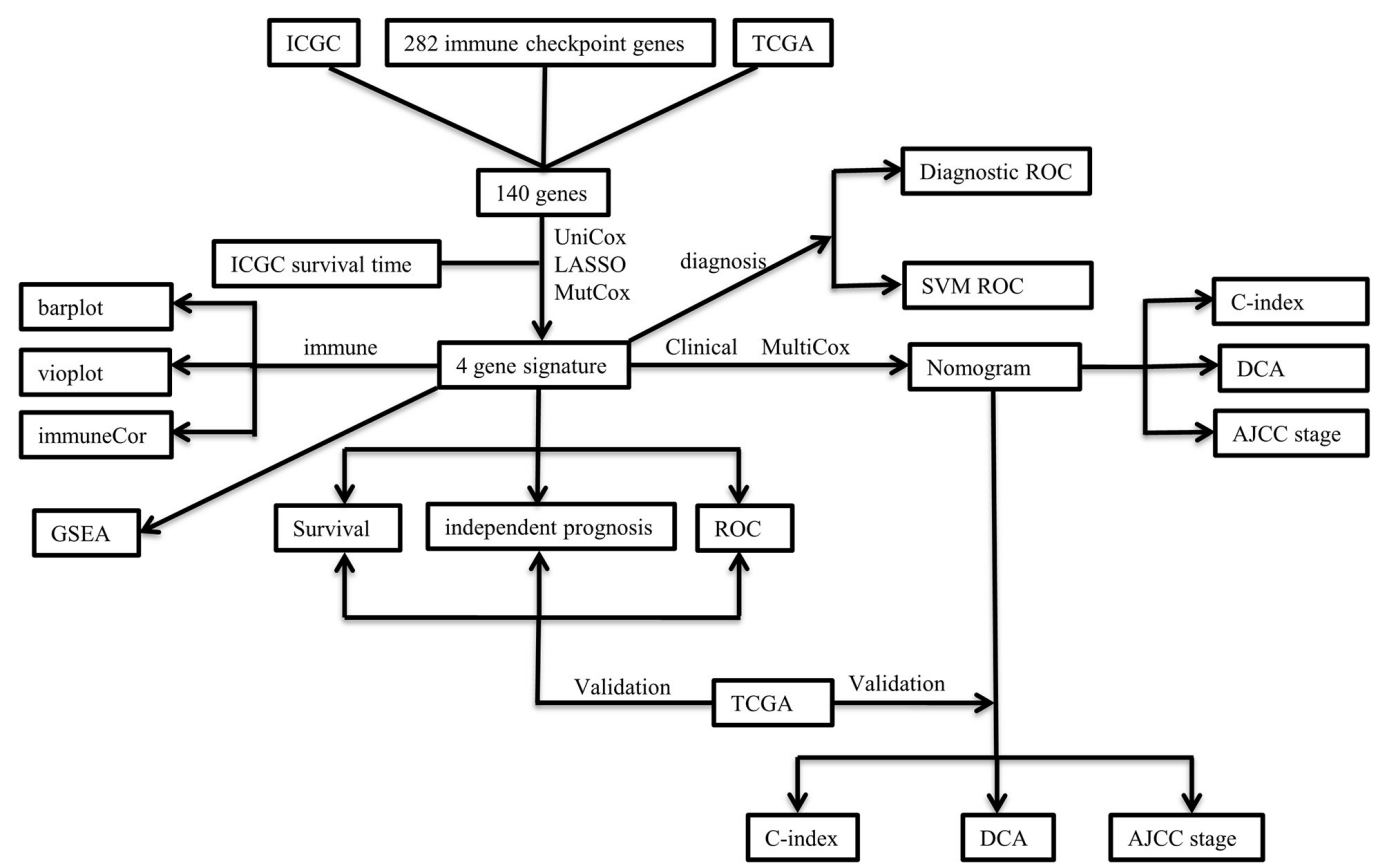
Flowchart of the research procedure in this study.

The clinicopathological characteristics of the TCGA and ICGC cohorts are listed in Table 1. Samples with clinicopathological and follow-up information were included for survival analysis in this study, consisting of 232 HCC samples in the ICGC cohort and 370 HCC samples in the TCGA cohort, respectively. The patient selection scheme and workflow chart are shown in Figure 1.

**Table 1:** Clinical data of patients in the ICGC and TCGA validation cohorts.

**Variables**	**Subgroups**	**ICGC (*n* = 232)**	**TCGA (*n* = 370)**
**Age**			
	<60 years	45	169
	≥60 years	187	201
**Sex**			
	Male	171	249
	Female	61	121
**Stage**			
	I	36	171
	II	106	85
	III	71	85
	IV	19	5
	Unknown	0	24
**Grade**			
	I	—	55
	II	—	177
	III	—	121
	IV	—	12
	Unknown	—	5
**Survival status**			
	Dead	43	130
	Living	189	240
**Vascular invasion**			
	Positive	—	108
	Negative	—	206
	Unknown	—	56
**Family history**			
	Positive	74	112
	Negative	143	207
	Unknown	15	51
**Prior malignancy**			
	Positive	30	35
	Negative	202	335
	Unknown	0	0

*ICGC, International Cancer Genome Consortium; TCGA, The Cancer Genome Atlas*.

### Feature Gene Identification and Prognostic Gene Signature Construction

**Figure 2:**
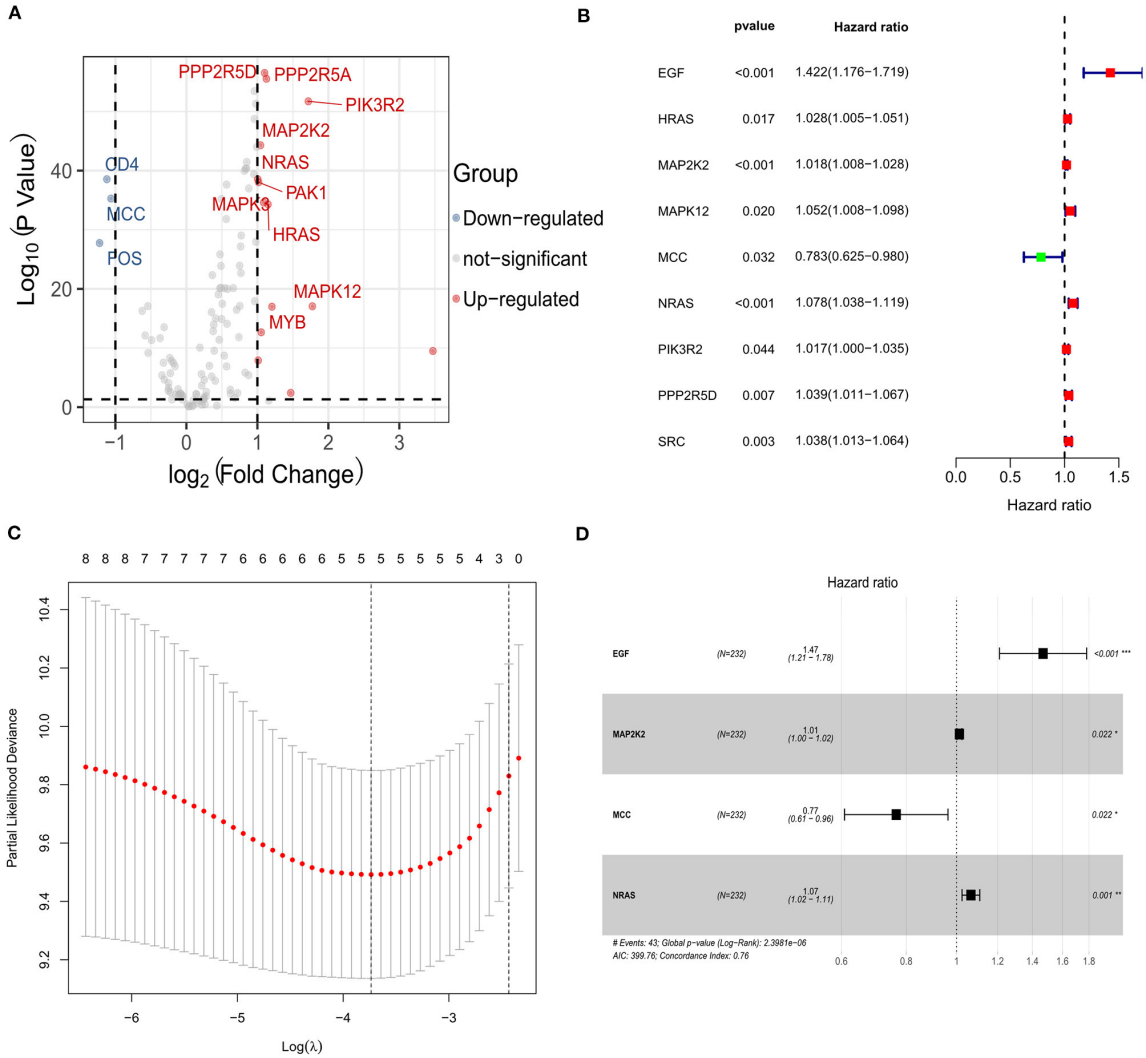
Identification of the candidate immune checkpoint–related genes in the ICGC cohort. **(A)** The volcano plot of the differentially expressed genes between hepatocellular carcinoma and adjacent normal samples. **(B)** Univariate Cox regression analysis identifying prognostic variables with HR with 95% CI and *P* values. **(C)** Selecting the tuning parameters for the LASSO regression algorithm. **(D)** Forest plots illustrating the associations of identified four immune checkpoint–related genes with OS in the ICGC cohort.

A total of 140 overlapping immune checkpoint–related genes between two cohorts were identified for subsequent analysis. Next, 14 up-regulated genes and 3 down-regulated genes were identified (Figure 2A). Afterward, univariate Cox analysis identified seven genes associated with survival (Figure 2B), and five genes retained after LASSO Cox regression (Figure 2C). Finally, multivariate Cox regression analysis was carried to build a risk signature. As a result, epidermal growth factor (EGF), MAP2K2, MCC (mutated in colorectal cancer), and NRAS (neuroblastoma RAS viral oncogene homolog) were determined as remarkably prognostic-related genes (Figure 2D). The risk score of the signature for each sample was calculated as the following equation: risk score = 0.384204567 ^*^ expression of EGF + 0.012818859 ^*^ expression of MAP2K2 + 0.063749656 ^*^ expression of NRAS – 0.267497698 ^*^ expression of MCC. Among them, EGF, MAP2K2, and NRAS had coefficients >0 and were considered high-risk factors associated with short survival; MCCs had coefficient <0 and were considered protective factors associated with long survival. The risk score was computed for each individual in the ICGC and TCGA cohort, and patients were classified into low- and high-risk groups.

### The Performance of Gene Signature

HCC patients of high-risk group showed a significantly unfavorable OS than patients of low-risk group in the ICGC cohort [hazard ratio (HR) = 5.83, 95% CI = 2.68–12.66, *P* < 0.0001; Supplementary Figure 1A] and further validated in the TCGA cohort (HR = 1.83, 95% CI = 1.28–2.61, *P* = 0.0009; Supplementary Figure 1B). Subsequently, to explore the stability and reliability of the signature, survival analysis in different subgroups was performed. As shown in Supplementary Figures 1C–L, the Kaplan–Meier curves demonstrated that the signature was a stable prognostic biomarker for patients with HCC stratified by age (<60 or ≥60 years), sex (male or female), stage (stage I–II or stage III–IV), cancer family history (yes or no), and without prior malignancy.

**Figure 3:**
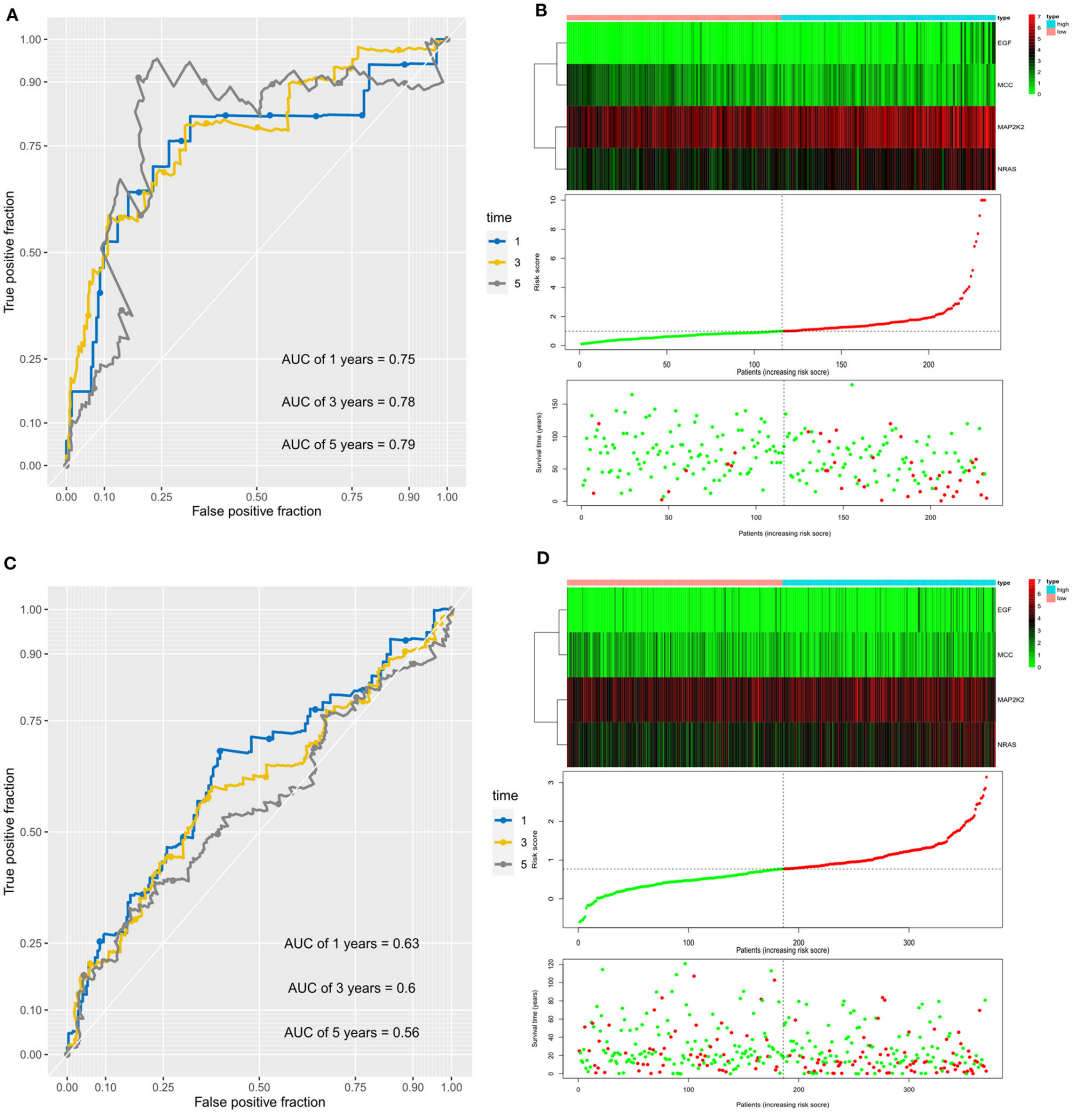
Prognostic value of the four immune checkpoint–related gene signature for prediction of overall survival of patients with hepatocellular carcinoma in the ICGC cohort. **(A)** ROC curve analysis for predicting survival in HCC patients according to the risk score in the ICGC cohort. **(B)** From top to bottom are the risk score, patients' survival status distribution, and the expression heatmap of four prognostic immune checkpoint–related genes in the low- and high-risk groups in the ICGC cohort. **(C)** ROC curve analysis for predicting survival in HCC patients according to the risk score in the TCGA cohort. **(D)** From top to bottom are the risk score, patients' survival status distribution, and the expression heatmap of four prognostic immune checkpoint–related genes in the low- and high-risk groups in the TCGA cohort.

In addition, the AUC values of the prognostic signature for the 1-, 3-, and 5-year survival rates in the ICGC cohort were 0.75, 0.78, and 0.79, respectively, (Figure 3A). The signature expression between two cohorts, risk score distribution, and survival status of each patient is shown in Figure 3B. The prognostic signature could separate HCC patients into low- and high-risk groups, and with the increasing risk score, patients in the ICGC cohort have a worse OS; the expression of prognostic genes increased. Moreover, the AUC values for OS in the TCGA cohort at 1, 3, and 5 years were 0.63, 0.60, and 0.56, respectively (Figure 3C). An increased risk score was associated with higher patient death rate (Figure 3D). These results confirmed that the novel signature accurately predicted the prognosis of HCC patients.

### Independent Prognostic Value of the Immune Checkpoint–Related Gene Signature

A multivariate Cox regression analysis was first performed among the available clinicopathological variables to determine whether the risk score was an independent prognostic factor for OS in the ICGC cohort. It was revealed that the risk score of the signature was significantly associated with the OS of patients with HCC after correction for other confounding factors variables (*P* < 0.0001, Table 2). Furthermore, after correction for other confounding factors in the TCGA cohort, the risk scores were still independent of other risk variables for OS in the multivariate Cox regression analysis (*P* = 0.043).

**Table 2:** Multivariate analyses identified independent prognostic factors for overall survival of HCC in the TCGA and the ICGC cohorts.

	**Multivariate analysis**		
	**HR**	**95% CI**	***P* value**
**ICGC cohort**			
Risk score	1.108	1.064–1.154	<0.0001
Age	0.976	0.942–1.011	0.1831
Sex	0.348	0.178–0.679	0.002
Stage	2.316	1.583–3.391	<0.0001
Prior cancer	2.586	1.031–6.489	0.0429
Family history of cancer	0.851	0.435–1.667	0.6386
**TCGA cohort**			
Risk score	1.539	1.012–2.339	0.0438
Age	1.017	0.996–1.038	0.1185
Sex	0.739	0.446–1.224	0.2402
Stage	1.311	0.972–1.769	0.0763
Grade	1.204	0.835–1.736	0.3193
Prior cancer	1.545	0.684–3.489	0.2952
Neoplasm status	1.631	0.972–2.735	0.0639
Vascular invasion	1.055	0.617–1.806	0.8443
Family history of cancer	1.105	0.667–1.832	0.6984

*ICGC, International Cancer Genome Consortium; TCGA, The Cancer Genome Atlas; HR, hazard ratio; CI, confidence interval*.

### Building and Validating a Predictive Nomogram Based on the Signature

As prior cancer, sex, stage, and the 4-gene signature were revealed to be independent prognostic variables for HCC, a nomogram incorporating these factors was built to predict 1-, 3-, and 5-year OS (Supplementary Figure 2A). The C-index in the ICGC cohort for prior cancer, sex, TNM stage, risk score, and the nomogram (combined model) was 0.526 (95% CI, 0.468–0.584), 0.567(95% CI, 0.488–0.646), 0.731 (95% CI, 0.675–0.787), 0.752 (95% CI, 0.664–0.840), and 0.766 (95% CI, 0.697–0.834), respectively. As for the TCGA cohort, C-index for prior cancer, sex, TNM stage, risk score, and the nomogram was 0.525 (95% CI, 0.481–0.569), 0.549(95% CI, 0.485–0.613), 0.567 (95% CI, 0.503–0.631), 0.621 (95% CI, 0.554–0.688), and 0.641 (95% CI, 0.577–0.705), respectively. Therefore, these findings demonstrated that compared with nomograms constructed with a single prognostic variable, the nomogram established with the combined model might be the optimal nomogram for predicting OS for patients with HCC, which might contribute to clinical management. Calibration plots showed that the nomogram prediction of survival probability was closely in agreement with the ideal prediction both in ICGC cohort (Supplementary Figure 2B) and TCGA cohort (Supplementary Figure 2C). According to decision curve analyses, the nomogram also offered the highest net benefit than the TNM stage examined in both cohorts (Supplementary Figures 2D,E).

### The Novel Gene Signature for Diagnostic Prediction of HCC

**Figure 4:**
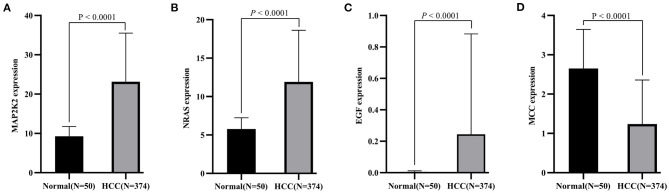
Validation of expression pattern of four identified genes (**A**, MAP2K2; **B**, NRAS; **C**, EGF, and **D**, MCC) in the TCGA cohort.

**Figure 5:**
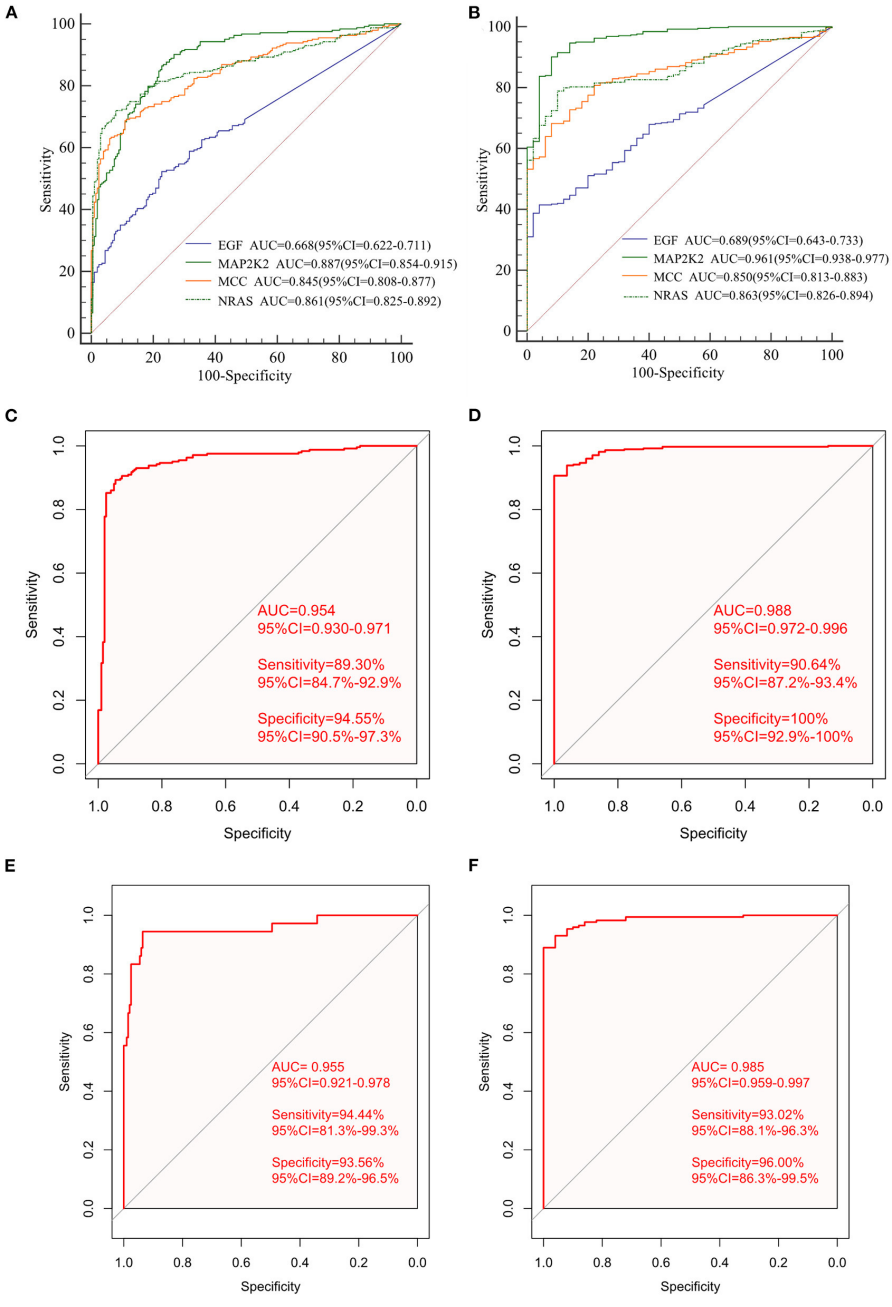
The diagnostic performance of signature in distinguishing HCC from normal samples. The ROC curves of single gene in the signature in the ICGC cohort **(A)** and the independent TCGA validation cohort **(B)**; ROC curves of the diagnostic prediction model for the ICGC cohort **(C)** and TCGA validation cohort **(D)**. ROC curves of the diagnostic prediction model for stage I patients with HCC in the ICGC cohort **(E)** and the TCGA cohort **(F)**.

First, the levels of expression of four feature genes were verified in the TCGA cohort. Consistent with the results in the ICGC cohort, MAP2K2, NRAS, and EGF were found to be significantly up-expressed, whereas MCCs were significantly down-expressed in HCC (all *P* < 0.0001, Figures 4A–D). In the early diagnosis of HCC, there is a need for sensitive and specific diagnostic biomarkers to accurately distinguish HCC patients from adjacent tissues. Next, ROC analysis was carried out to investigate the diagnostic performance of the four genes for HCC between 240 HCC and 202 adjacent samples in the ICGA cohort. As revealed in Figure 5A, AUCs for EGF, MAP2K2, MCC, and NRAS were 0.668 (97.5% CI, 0.622–0.711), 0.887 (97.5% CI, 0.854–0.915), 0.845 (97.5% CI, 0.808–0.877), and 0.861 (97.5% CI, 0.825–0.892). In addition, the diagnostic power of single gene in the signature was further confirmed in the TCGA cohort (Figure 5B). AUCs for EGF, MAP2K2, MCC, and NRAS were 0.689 (97.5% CI, 0.643–0.733), 0.961 (97.5% CI, 0.938–0.977), 0.850 (97.5% CI, 0.813–0.883), and 0.863 (97.5% CI, 0.826–0.894). We further used SVM algorithm to establish a diagnostic model that integrated the four immune checkpoint–related genes to distinguish between HCC patients and adjacent tissues. The diagnostic model demonstrated a perfect diagnosis performance with AUC of 0.954 (95% CI, 0.930–0.971), sensitivity of 89.30% (95% CI, 84.7%−92.9%), and specificity of 94.55% (95% CI, 90.5%−97.3%) to distinguish HCC from adjacent tissues in the ICGC cohort (Figure 5C). What is more, the powerful diagnostic capacity was further validated in the TCGA cohort (Figure 5D). The model illustrated perfect discriminatory performance in the diagnosis of HCC with AUC of 0.988 (95% CI, 0.972–0.996), sensitivity of 90.64% (95% CI, 87.2%−93.4%), and specificity of 100% (95% CI, 92.9%−100%) to distinguish HCC from adjacent tissues. Particularly, the diagnostic model illustrated a perfect diagnosis performance for early stage of HCC (TNM stage I), as revealed in Figure 5E.

The diagnostic model diagnosed HCC patients at early stage with a sensitivity of 94.44%, specificity of 93.56%, and AUC of 0.955. We then validated the diagnostic model using the TCGA cohort. The diagnostic model diagnosed HCC patients at early stage with a sensitivity of 93.02%, specificity of 96.0%, and AUC of 0.985 (Figure 5F).

### Gene Set Enrichment Analyses

The GSEA was carried out to reveal the biological processes altered between high-risk and low-risk groups. As revealed in the Supplementary Figure 3A, cell cycle, DNA replication, extracellular matrix (ECM)–receptor interaction, bladder cancer, gastric cancer, non–small cell lung cancer, and mismatch repair pathways were significantly enriched in the high-risk group. Chemical carcinogenesis, cytokine–cytokine interaction, JAK-STAT signaling pathway, and PPAR signaling pathway were significantly enriched in the low-risk group (Supplementary Figure 3B).

### Immune Cell Infiltration and the Association With Four Immune Checkpoint–Related Genes

**Figure 6:**
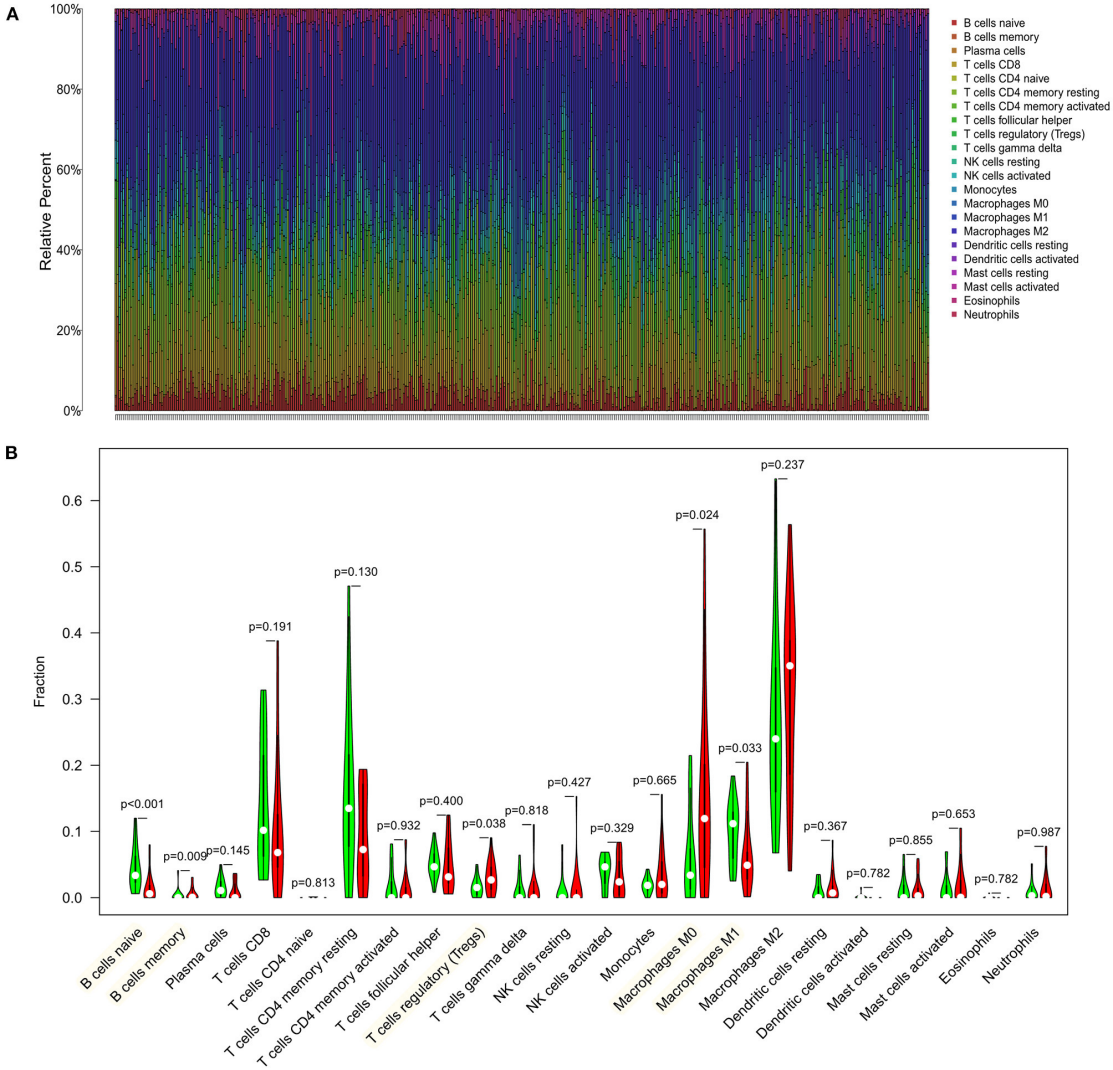
Distribution and visualization of immune cell infiltration in HCC patients. **(A)** Summary of estimated compositions of 22 immune cell subtypes from the CIBERSORT algorithm. **(B)** Comparison of 22 immune cell subtypes between low- and high-risk samples. Blue and red colors represent low- and high-risk samples, respectively.

**Figure 7:**
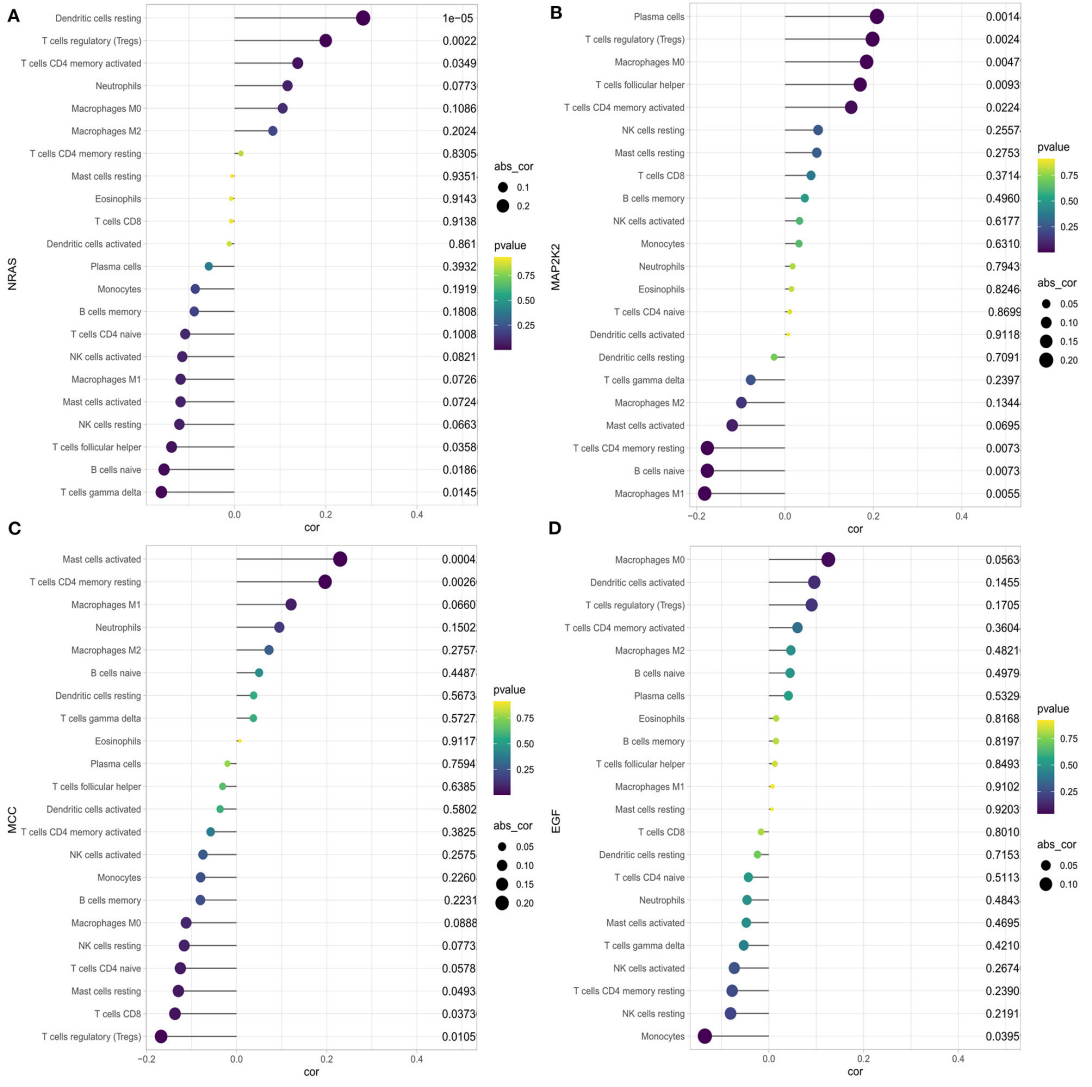
Correlation between NRAS **(A)**, MAP2K2 **(B)**, MCC **(C)**, EGF **(D)**, and infiltrating immune cells in patients with HCC.

We first explored the composition of immune cells in HCC patients (Figure 6A). The proportions of regulatory T cells (Tregs), macrophages M0, and macrophages M1 in the high-risk group were significantly higher than in the low-risk group (all *P* < 0.05, Figure 6B). However, the proportion of naive cells (*P* < 0.001) and memory cells (*P* = 0.009) in high-risk group were significantly lower than in low-risk group. As revealed in the Figure 7A, NRAS was positively correlated with resting dendritic cells, Tregs, and activated CD4 memory T cells (all *P* < 0.05) and negatively correlated with follicular T helper cells, naive B cells, and gamma delta T cells (all *P* < 0.05). MAP2K2 was positively correlated with plasma cells, Tregs, macrophages M0, follicular helper T cells, and activated CD4 memory T cells (all *P* < 0.05) and negatively correlated with CD4 memory resting T cells, naive B cells, and M1 macrophages (Figure 7B; all *P* < 0.05). MCC was positively correlated with activated mast cells and CD4 memory resting T cells (all *P* < 0.05) and negatively correlated with CD8 T cells, Tregs, resting mast cells (Figure 7C; all *P* < 0.05). EFG was negatively correlated with monocytes (Figure 7D; *P* = 0.0395).

### Potential of the Risk Score as an Indicator of Response to Immunotherapy

The association between the risk score and expression levels of three immune checkpoint genes was explored. As shown in Supplementary Figure 4, the risk score was significantly positively correlated with CTLA4 (coefficient = 0.114, *P* = 0.029) and showed a positive trend for PD-L1 (coefficient = 0.092, *P* = 0.078). However, it was not significantly correlated with PD1 (*P* = 0.575). TMB along with copy-number alteration can be used to categorize cancers into distinct sensitivity to immune checkpoint inhibitor therapy across pan-cancers (Liu et al., 2019). We further demonstrated that patients in high-risk group presented a significantly higher TMB than patients in the low-risk group (*P* = 0.0329), revealing that the high-risk group was more likely to have an immune response and response to immunotherapy.

## Discussion

HCC is one of the most prevalent deadly malignancies worldwide, showing a poor prognosis due to the high molecular and cellular heterogeneity and high rate of recurrence and metastasis (Desai et al., 2017; Finn et al., 2018). Although great and rapid progress has been made in surgical and medical therapy methods, the prognosis of HCC remains unsatisfactory. Lack of efficient detection biomarkers on the early stage contributes to the progression of HCC, and survival times differ greatly even among patients with the same TNM stage of disease. Therefore, early diagnostic markers and novel accurate prognostic models are urgently required in diagnosis and predict the survival of HCC patients.

In the past few decades, critical breakthroughs have been made in the field of immune surveillance, including the involvement of PD-1/PD-L1 and CTLA-4 signaling pathways in the development and progression of cancers, which plays a vital role in the regulation of immune responses (Wu et al., 2020). CTLA-4 is the first checkpoint protein blockade proven to be effective in cancer immunotherapy. It can migrate to the surface of T cells and compete with CD28 for binding to CD80 and CD86, thus inhibiting the proliferation and activation of T cells (Jiang et al., 2019). Moreover, other immune checkpoint molecules, such as PD-1, PD-L1, CD28, galectin-9 (Gal-9), and T cell immunoglobulin and mucin domain 3 (TIM-3), can properly regulate the immune system to avoid autoimmune responses caused by excessive activated immune cells (Zou et al., 2016). Nevertheless, when the immune checkpoint genes are overexpressed or activated, the immune function will be inhibited. As a result, cancer cells that excessively activate immune checkpoint genes can prevent local immune cells from escaping surveillance and clearance, thereby accelerating its growth (Wu et al., 2020).

However, the number of immune checkpoint–related gene biomarkers and prognostic models that could be utilized to predict the survival of HCC patients is still lacking. The present study aimed to identify an effective prognostic signature to stratify HCC patients and predict the survival of HCC. In this study, a total of 140 shared immune checkpoint–related genes were identified from two datasets. Five prognosis-related DEGs were screened out by using univariable Cox regression and LASSO algorithms in the ICGC cohort, which were then subjected to multivariate Cox regression analysis. Finally, a novel four-gene signature was generated and validated its efficiency in TCGA cohort, which could successfully categorize patients into low- and high-risk groups with distinct OS, where the high-risk subset exhibited a significantly poor prognosis pattern than the low-risk group. The efficacy of novel signature was found in the development cohort, validating cohort, and the subgroup from ICGC, indicating a robust high prognostic value of the signature. The AUC values of the prognostic signature for OS prediction presented excellent predictive performance in both cohorts. This four-gene signature also demonstrated to be an independent prognosis factor for HCC survival in two cohorts. A nomogram combining gender, prior malignancy, tumor stage, and risk score was proposed, which proved to be a better predictor than nomograms constructed with a single prognostic variable. The nomogram constructed with the combined model might be the optimal model for predicting OS for patients with HCC, which contributes to clinical management of HCC. Next, the SVM classifier incorporating the four genes displayed perfect discriminatory ability in distinguishing HCC from adjacent tissues with an AUC of 0.954 (95% CI, 0.930–0.971) in the ICGC cohort and an AUC of 0.988 (95% CI, 0.972–0.996) in the TCGA cohort. Furthermore, the diagnostic model illustrated a perfect diagnosis performance for early stage of HCC with an AUC of 0.955 (95% CI, 0.921–0.978) in the ICGC cohort and validated in the TCGA cohort with an AUC of 0.985 (95% CI, 0.959–0.997). We utilized a more comprehensive approach to develop a robust prognostic signature for HCC and successfully validated it in an external cohort. Moreover, high-risk group was more likely to have an immune response and respond to immunotherapy. Therefore, this immune checkpoint–related gene prognostic signature is accurate, robust, and interpretable. Tumor-infiltrating immune cells have a high prognostic value as to tumor progression and patient's survival in many solid organ malignancies (Marabelle et al., 2014). We found that the four genes were correlated with multiple immune cells; for example, NRAS was correlated with dendritic cells resting, Tregs, CD4 memory activated T cells, follicular helper T cells, naive B cells, and gamma delta T cells.

We identified four risky prognostic genes (EGF, MCC, MAP2K2, and NRAS). The EGF protein acts by binding with high affinity to the cell surface receptor and epidermal growth factor receptor (EGFR). Dysregulation of EGF has been correlated with the development and progression of multiple malignancies. Previous study had been validated that epithelial–mesenchymal transition (EMT) in cancer cells is a vital step in malignancy progression, including cancer growth, invasion, and metastasis, and contributes to a high malignancy stage (Lindsey and Langhans, 2014). EGF is one of the growth factors and is known to play a role in EMT in HCC (Lim et al., 2020). EMTs and their associated early metastasis-related processes are activated by multiple growth factors such as EGF, transforming growth factor β, and platelet-derived growth factor (Gurzu et al., 2019). In addition, previous animal studies have demonstrated that targeted overexpression of EGF induces the formation of highly malignant HCC in mice, and its receptor EGFR is also up-expressed in HCC tissues (Liu et al., 2018). Interferon γ (IFN-γ), EGFR, and mitogen-activated protein kinase (MAPK) signaling pathways were associated with PD-L1 gene expression in HCC. EGF stimulation enhanced PD-L1 mRNA and protein expression levels in a representative HCC cell line group, further increased by EGF and IFN-γ stimulation (Xing et al., 2020). MCC, which located on chromosome 5q21 and encoded a protein that comprised 829 amino acids, is a candidate colorectal cancer suppressor gene that is reported to negatively modulate cell growth and differentiation and cell cycle and could suppress Wnt/β-catenin signal transduction (Fukuyama et al., 2008; Wang et al., 2016). MCC functions as an oncogene in B cells and may serve as a diagnostic marker and therapeutic target in B cell malignancies (Edwards et al., 2014). As a member of the MAPKK/MAP2K family, mitogen-activated protein kinase kinase 2 (MAP2K2) was demonstrated to be correlated with tumorigenesis and involved in the well-known RAS-RAF-MAP2K/MEK-MAPK/ERK pathway (Codogno and Meijer, 2005; Shinojima et al., 2007). A new mutation in MAP2K2 gene was reported, which most likely conferred resistance to dabrafenib and trametinib treatment and anti-PD1 therapies (nivolumab plus pembrolizumab), whereas a frameshift mutation in B2M was the strongest candidate alteration for progression on checkpoint inhibitor therapy in melanoma (Richmond et al., 2019). A previous transcriptome profiling study demonstrated that neuroblastoma RAS viral oncogene homolog (NRAS) was dysregulated in fibrolamellar HCC; however, the possible clinical implications or the function of NRAS has not been explored (Sorenson et al., 2017). Additionally, NRAS overexpression associated with poor outcome and proliferation *in vivo*. NRAS knockdown enhanced sorafenib efficacy in resistant cells and may be a prognostic predictor in HCC (Dietrich et al., 2019).

It was reported that NRAS mutations and PD-L1 expression were most common in primary vaginal melanomas and can be probably used as therapeutic targets (Wang et al., 2020). Meanwhile, the results of GSEA analysis illustrated that the four-gene signature-enriched pathway was notably associated with tumorigenesis pathways and immune-related biological processes.

To our knowledge, this is the first study to establish a prognostic signature based on immune checkpoint–related genes in HCC. However, our study had some limitations. In this study, some other important variables were unviable such as history of cirrhosis, history of hepatitis B virus, α-fetoprotein value, alcohol consumption, etiology of liver disease, and the main mode of treatment, which may have had a certain effect on the results. In addition, further validation of the effectiveness of the signature in other independent prospective studies and functional experiments of the identified genes is needed. Besides, more prospective clinical trials with larger sample sizes are required for further evaluation the potential diagnostic power. Thus, there is still a long way to go before the findings can be applied to clinical practice.

## Conclusion

A novel immune checkpoint–related gene signature was developed, and it presented great potential clinical application value in predicting the OS for patients with HCC. The signature could act as a robust biomarker for early diagnostic and prognostic of HCC.

## Data Availability Statement

Publicly available datasets were analyzed in this study. This data can be found here: The data sets involved in our study are publicly available in ICGC database (https://dcc.icgc.org/) and the TCGA database (https://portal.gdc.cancer.gov/).

## Author Contributions

SC and YD is the principle investigator. EZ conducted statistical analysis and data management. EZ edited and SC wrote and revised the manuscript. All authors read and approved the final manuscript.

## Conflict of Interest

The authors declare that the research was conducted in the absence of any commercial or financial relationships that could be construed as a potential conflict of interest.

## Supplementary Material

Supplementary Figure 1 Kaplan-Meier survival analysis of gene signature between the high- and low-risk groups. Survival differences in the ICGC cohort **(A)**; and TCGA validation cohort **(B)**; Survival curves stratified by age≥60 **(C)**, age < 60 **(D)**, female **(E)**, male **(F)**, stage I-II **(G)**, stage III-IV **(H)**, with family history of cancer **(I)**, without family history of cancer **(J)**, without prior malignancy **(K)**, and with prior malignancy **(L)**.

Supplementary Figure 2 Nomogram construction and validation. **(A)** Nomogram predicting overall survival probability for patients with HCC in the ICGC cohort. **(B)** Calibration plots for the nomogram in both cohorts. **(C)** decision curve analyses comparing nomogram and AJCC stage in both cohorts.

Supplementary Figure 3 The significantly altered biological processes in high-risk patients in ICGC cohort by GSEA.

Supplementary Figure 4 The association between three immune checkpoint genes, tumor mutation burden (TMB) and signature risk score. Correlation analysis between CTLA4 **(A)**, PD1 **(B)**, PD-L1 **(C)** and risk score; Boxplots of TMB distribution in the high- and low-risk groups stratified by the risk score **(D)**.

Supplementary Table 1 A total of 282 unique immune checkpoint-related genes.
