# Supplementary figures and images for: Development and External Validation of a Novel Immune Checkpoint–Related Gene Signature for Prediction of Overall Survival in Hepatocellular Carcinoma
Source: Front Mol Biosci. 2021 Jan 21;7:620765. doi: 10.3389/fmolb.2020.620765 (PMC7859359; doi:10.3389/fmolb.2020.620765)

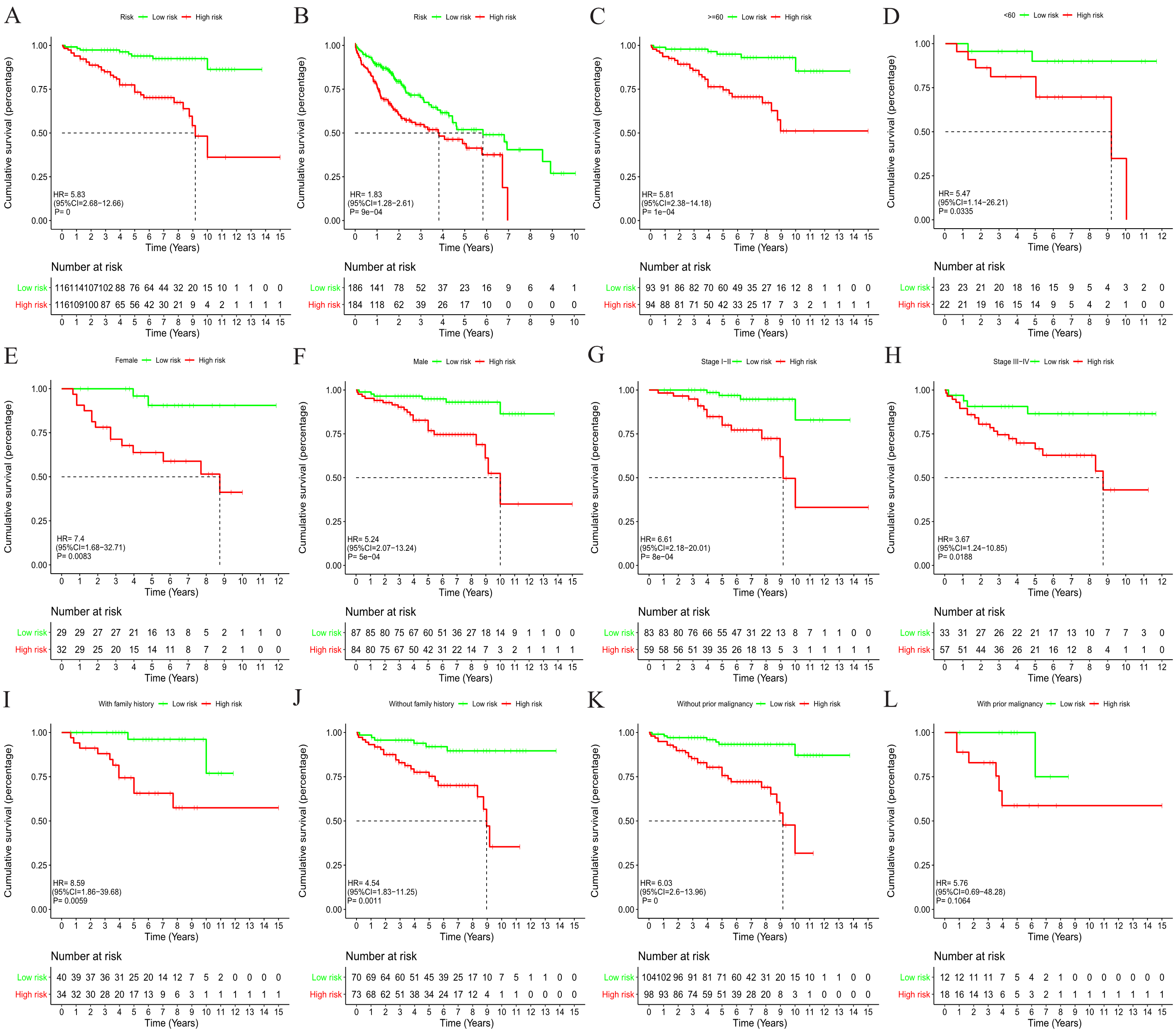

Supplement: Supplementary Figure 1 — Kaplan-Meier survival analysis of gene signature between the high- and low-risk groups. Survival differences in the ICGC cohort (A); and TCGA validation cohort (B); Survival curves stratified by age≥60 (C), age < 60 (D), female (E), male (F), stage I-II (G), stage III-IV (H), with family history of cancer (I), without family history of cancer (J), without prior malignancy (K), and with prior malignancy (L). [file Image_1.TIFF]

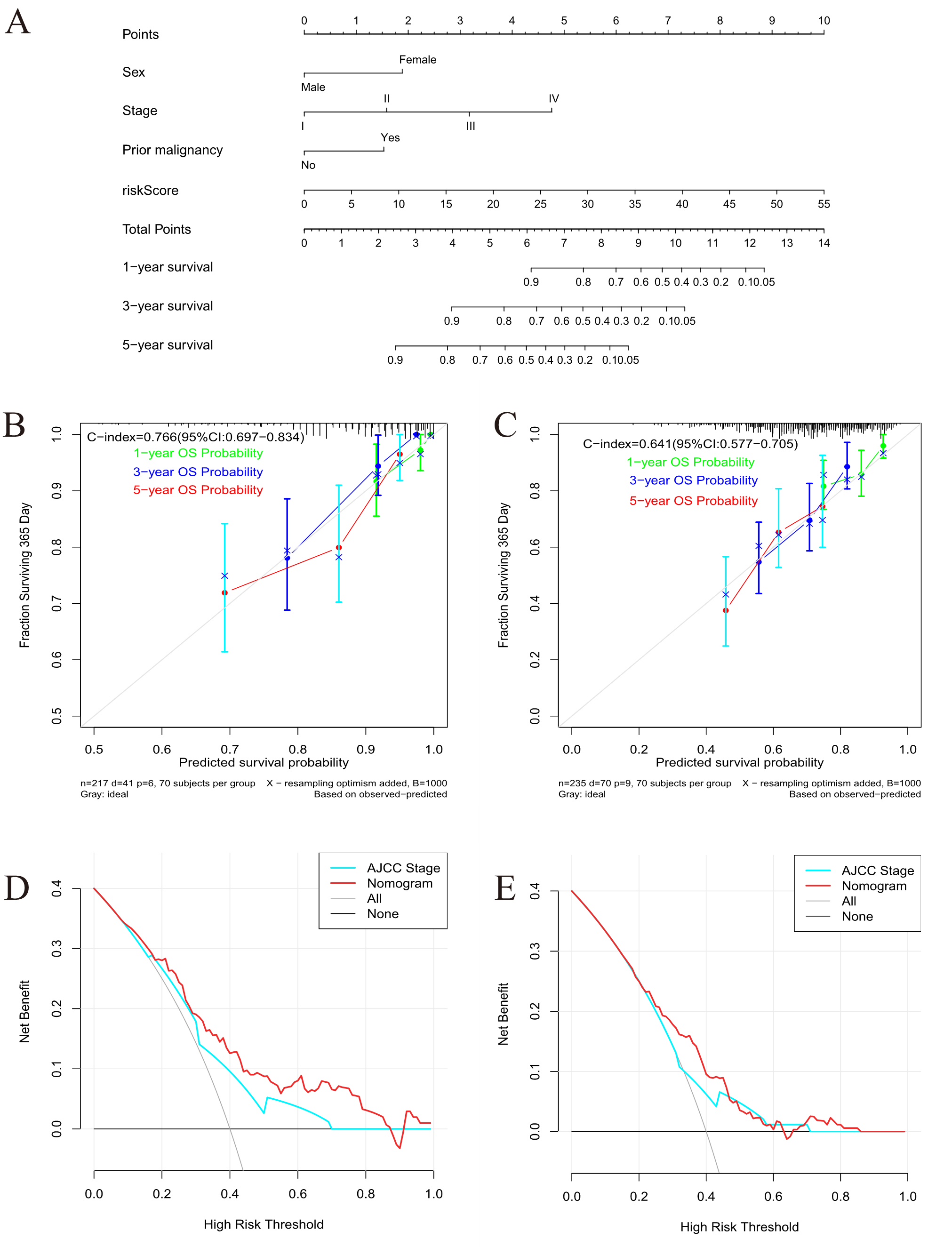

Supplement: Supplementary Figure 2 — Nomogram construction and validation. (A) Nomogram predicting overall survival probability for patients with HCC in the ICGC cohort. (B) Calibration plots for the nomogram in both cohorts. (C) decision curve analyses comparing nomogram and AJCC stage in both cohorts. [file Image_2.tiff]

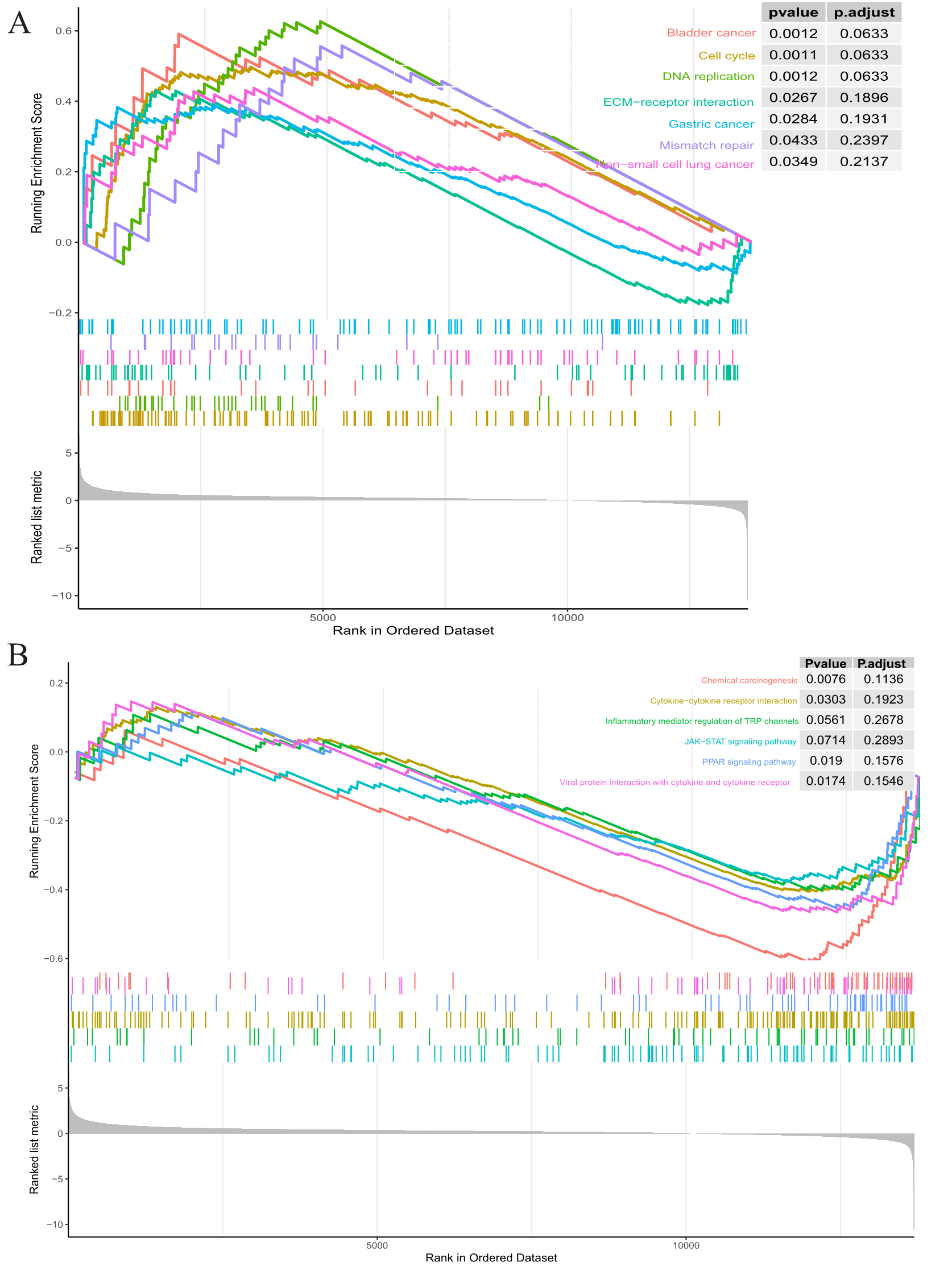

Supplement: Supplementary Figure 3 — The significantly altered biological processes in high-risk patients in ICGC cohort by GSEA. [file Image_3.TIFF]

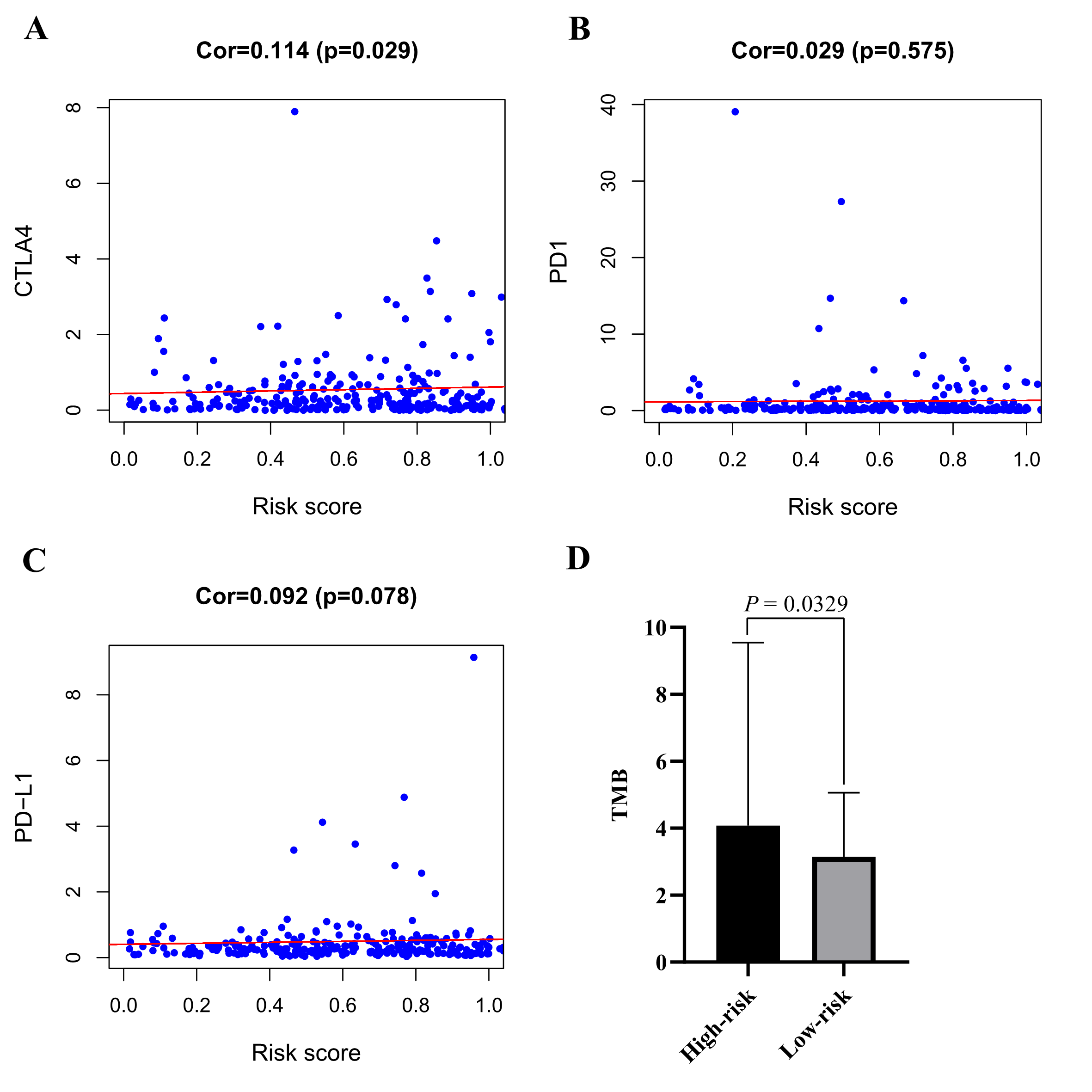

Supplement: Supplementary Figure 4 — The association between three immune checkpoint genes, tumor mutation burden (TMB) and signature risk score. Correlation analysis between CTLA4 (A), PD1 (B), PD-L1 (C) and risk score; Boxplots of TMB distribution in the high- and low-risk groups stratified by the risk score (D). [file Image_4.TIFF]
