# Supplementary material for: Development and External Validation of a Novel Immune Checkpoint–Related Gene Signature for Prediction of Overall Survival in Hepatocellular Carcinoma
Source: Front Mol Biosci. 2021 Jan 21;7:620765. doi: 10.3389/fmolb.2020.620765 (PMC7859359; doi:10.3389/fmolb.2020.620765)
Supplement: Supplementary Table 1 — A total of 282 unique immune checkpoint-related genes. [file Table_1.DOCX]

**Supplementary Table S1** A total of 282 unique immune checkpoint-related genes

| AAM | BIU | CFR | DRO | GGO | ICOS | LYN | MGP | NLE | PDCD1LG2 |
| --- | --- | --- | --- | --- | --- | --- | --- | --- | --- |
| ACS | BOM | CGE | EAI | GJA | ICOSLG | MALB | MJV | NMEL | PDPK1 |
| ACUN | BTA | CHUK | ECB | GRAP2 | IFNG | MAP2K1 | MLST8 | NNI | PHI |
| ACYG | BTLA | CHX | EEE | GRB2 | IFNGR1 | MAP2K2 | MMU | NRAS | PHYP |
| AJU | CCAE | CJC | EGF | HAI | IFNGR2 | MAP2K3 | MNA | OAA | PIK3CA |
| AKT1 | CCAN | CJO | EGFR | HGL | IKBKB | MAP2K6 | MPAH | OAS | PIK3CB |
| AKT2 | CCAR | CLV | EGZ | HIF1A | IKBKG | MAP3K14 | MTOR | OCU | PIK3CD |
| AKT3 | CCW | CMK | ELK | HLA-DPA1 | IPU | MAP3K3 | MUN | OLA | PIK3R1 |
| ALK | CD247 | CMY | ELS | HLA-DPB1 | JAK1 | MAP3K8 | MYB | ONL | PIK3R2 |
| AMEX | CD274 | CPIC | EML4 | HLA-DQA1 | JAK2 | MAPK1 | MYD | OOR | PIK3R3 |
| AMJ | CD28 | CSAB | EPZ | HLA-DQA2 | JUN | MAPK11 | MYD88 | ORO | PKI |
| AML | CD3D | CSK | ETL | HLA-DQB1 | KMR | MAPK12 | MZE | OTW | PLCG1 |
| AOCE | CD3E | CSNK2A1 | FAB | HLA-DQB2 | KRAS | MAPK13 | NFATC1 | PADL | PMAJ |
| APLA | CD3G | CSNK2A2 | FCA | HLA-DRA | LAT | MAPK14 | NFATC2 | PAK1 | PMUA |
| ASN | CD4 | CSNK2A3 | FCH | HLA-DRB1 | LAV | MAPK3 | NFATC3 | PAK2 | PMUR |
| BACU | CD80 | CSNK2B | FOS | HLA-DRB3 | LCF | MAPKAP1 | NFKB1 | PAK3 | PON |
| BATF | CD86 | CTLA4 | FPG | HLA-DRB4 | LCK | MCAL | NFKBIA | PALE | POV |
| BATF2 | CDC42 | CVG | FYN | HLA-DRB5 | LCM | MCC | NFKBIB | PBI | PPAD |
| BATF3 | CDK | DLE | GFR | HRAS | LSR | MCF | NFKBIE | PCAD | PPP2CA |
| BBUB | CFA | DRE | GGA | HSA | LVE | MDO | NGI | PCW | PPP2CB |
| ZAP70 | TRAV19 | YES1 | VVP | XCO | XLA | XMA | XTR | PDCD1 | PPP2R1A |
| PPP2R1B | VAV1 | UMR | UAH | TUP | TSR | TRIB3 | TRBV7-9 | TRBV12-3 | TRAV29DV5 |
| SLAL | SRC | SRX | SSC | STAT1 | STAT3 | TGU | THEM4 | TICAM1 | TICAM2 |
| RAC1 | RAF1 | RASGRP1 | RAY | RBB | RELA | RICTOR | RNO | RPS6KB1 | RPS6KB2 |
| RTP | SALP | SANH | SASA | SBQ | SCAN | SDU | SFM | SGH | SHR |
| TLR4 | TLR9 | TMU | TNFRSF14 | TRAC | TRAF6 | TLR2 | PVT | TRAV8-4 | TIRAP |
| PPP2R5A | PPP2R5B | PPP2R5C | PPP2R5D | PPP2R5E | PPP3CA | PPP3CB | PPP3CC | PPP3R1 | PPP3R2 |
| PPS | PRET | PRKCQ | PRR5 | PSS | PTEN | PTG | PTPN11 | PTPN6 | PTR |
| RRO | TRBC1 |  |  |  |  |  |  |  |  |
